# Long-Term Clinical Outcome after Treatment for High-Grade Cervical Lesions: A Retrospective Monoinstitutional Cohort Study

**DOI:** 10.1155/2015/984528

**Published:** 2015-06-09

**Authors:** Annarosa Del Mistro, Mario Matteucci, Egle Alba Insacco, GianLibero Onnis, Filippo Da Re, Lorena Baboci, Manuel Zorzi, Daria Minucci

**Affiliations:** ^1^Immunology and Molecular Oncology Unit, Veneto Institute of Oncology-(IOV-) IRCCS, Via Gattamelata 64, 35128 Padua, Italy; ^2^Department of Woman and Child Health, Azienda Ospedaliera di Padova, Via Giustiniani 2, 35128 Padua, Italy; ^3^Department of Pathology, Azienda Ospedaliera di Padova, Via Gabelli, 35121 Padua, Italy; ^4^Veneto Tumour Registry, Passaggio Gaudenzio 1, 35131 Padua, Italy; ^5^Department of Surgery, Oncology and Gastroenterology, University of Padua, 35128 Padua, Italy

## Abstract

*Background*. The aim of this retrospective observational study of women treated for cervical intraepithelial neoplasia grade 2 or worse (CIN2+) was to assess the long-term risk of residual/recurrent high-grade CIN. *Materials and Methods*. We evaluated 760 women treated by loop electrosurgical excision procedure (684) or conization (76) between 2000 and 2009, and followed up to June 30, 2014 (median follow-up 6.7 years, range 4–14). Visits every 6 months for the first year after treatment and yearly for up to the following 10 years included cytology, colposcopy when indicated, and HPV testing (search and typing). *Results*. CIN2+ or vaginal intraepithelial neoplasia grade 2 or worse (VAIN2+) was detected in 67 cases (8.8%), 39 at first follow-up and 28 after one/more negative visits. The risk of CIN2+ was higher in case of positive margins (odds ratio (OR) 8.04, 95% CI 4.31–15.0), type 3 transformation zone (OR for CIN3 27.7, 95% CI 2.07–36.9), CIN3+ excision (OR 6.02, 95% CI 1.73–20.9), and positive high-risk HPV test at first follow-up (OR for HPV16: 20.6, 95% CI 6.8–62.6; OR for other hrHPV types: 18.3, 95% CI 5.9–57.0). *Conclusion*. Residual/recurrent high-grade CIN occurred in <9% cases, and the risk was associated with transformation zone type, lesion grade, margins status, and hrHPV test result at 6–12 months of follow-up.

## 1. Introduction

Detection and treatment of preneoplastic high-grade lesions (cervical intraepithelial neoplasia grades 2 and 3 or worse (CIN2+)) can prevent the development of invasive cervical cancer and are the aim of the cervical cancer screening programmes. Different guidelines recommend treatment of all high-grade lesions since their risk of progression is approximately 30% [1, 2]. A proportion (estimated to be about 20–30% for CIN3 and 50–60% for CIN2 and even higher in young women) would undergo spontaneous regression if left untreated, but unfortunately no reliable biomarkers for discriminating regressive from progressive lesions have been identified to date [3].

The treatment should be effective in eradicating the high-grade lesion and have minimum morbidity and adverse effects on future fertility and pregnancy outcomes, particularly in young women. Conservative excisional methods are therefore the treatment of choice. Loop electrosurgical excision procedure (LEEP) and needle conization are the most widely used methods (especially in the last 10 years); the major advantages are specific tailoring of the treatment (which minimizes adverse effects) and histological evaluation of the treated lesion. These modalities show high efficacy in eradicating intraepithelial lesions, although failure rates of 5–30% are reported [4]. As a consequence of treatment failure, women treated for high-grade lesions have a risk of progression to invasive cancer 4-5 times higher than the general population [5–7]. This implies that follow-up procedures must be put in place and need to be effective in detecting residual/recurrent disease, while containing the number of visits for successfully treated women. Follow-up has been traditionally carried out by regular Pap smears, with or without colposcopy, for some years. In the last years, several studies have demonstrated that human papillomavirus (HPV) testing performed 6–12 months after treatment is a valuable tool: it is more sensitive than cytology in identifying women with residual/recurrent disease and has a very high negative predictive value (NPV) [8–12]. Search for high-risk HPV (hrHPV) types as a pool is the modality used in most studies, and specific typing is not recommended, although an increased risk of recurrence has been demonstrated for women treated for HPV16-associated high-grade lesions [13, 14]. Implementation of hrHPV testing in clinical practice depends on several factors and can take time before reaching a regular and consistent application.

The aim of the present study was to analyze the clinical outcome of 760 women treated for high-grade lesions and followed up for a minimum of 2 to more than 14 years, in order to assess the risk of residual/recurrent high-grade CIN and identify the best predictive indicators for recurrence and their role in the diagnostic strategy.

## 2. Patients, Materials, and Methods

### 2.1. Patients and Clinical Procedures

Subjects eligible for this retrospective monoinstitutional (Azienda Ospedaliera di Padova, Department of Woman and Child Health, Gynecology Service) analysis were women diagnosed with and treated for CIN2+ by loop electrosurgical excision procedure (LEEP) or conization by electric needle, having a follow-up of minimum 2 years.

All involved colposcopists performed regularly more than 500 procedures per year and had a total experience ranging from 20 to 40 years. Colposcopic features were recorded and described according to Barcelona nomenclature of the International Federation for Cervical Pathology and Colposcopy (IFCPC) [15]. Biopsies were performed on abnormal areas under colposcopic guide (directed biopsies); endocervical brushing was done in cases of not fully visible squamocolumnar junction (SCJ) or presence of atypical glandular cells (AGC) in the Pap smear, with curettage in cases of high-grade lesion and/or type 3 SCJ. Data on cytology, colposcopy, and diagnostic biopsies done before treatment were recorded.

**Figure 1:**
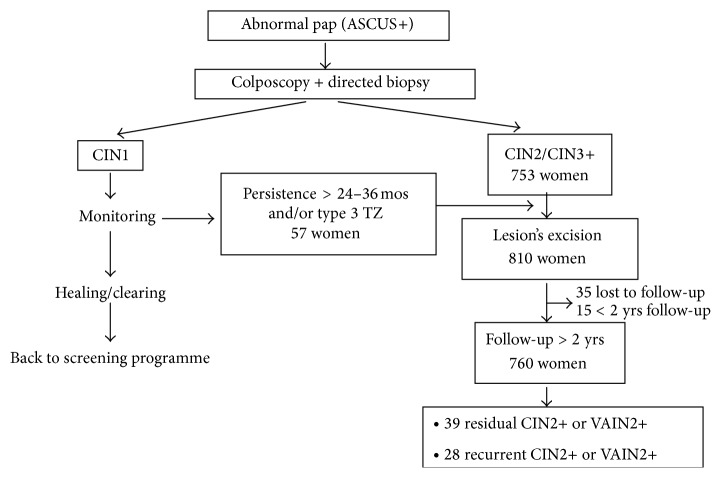
Flow chart of diagnostic work-up leading to lesion's treatment, and high-grade lesions detected during follow-up.

The diagnostic work-up leading to lesion's treatment is depicted in Figure 1.

LEEP and conization were performed under colposcopic guide in local anesthesia in an outpatient facility by experienced personnel [16]; loops of different sizes were used according to lesion's characteristics and cervix conformation; in all cases, care was taken to personalize the loop size. Excision margins were kept 2-3 mm out of the lesion, and completeness of lesion's removal was colposcopically verified.

At the time of treatment, the following data were collected: conformation and size of the cervix; transformation zone (TZ) type; size, grade, and number of the lesion(s) and their relation with endocervical canal and vagina; treatment modality; histology of the excised lesion (including margin section status), and patient's age.

Follow-up procedures included the following:

- cytology and colposcopy 6 and 12 months after treatment (an additional visit 3 months after treatment was recommended in cases with positive section margins or microinvasive carcinoma),
- HPV testing (search and typing) at 6 and 12 months after treatment which was added from 2005 and gradually implemented,
- cytology at yearly intervals for the following 10 years, with colposcopy in case of abnormal Pap.

Patients showing a cervical (CIN) or vaginal (VAIN) high-grade lesion at first follow-up visit after treatment were considered as having residual disease; patients showing a cervical or vaginal high-grade lesion after one or more follow-up visits with negative cytology and colposcopy were considered as having recurrent disease [17]. Patients with invasive lesions were considered as having progressive disease. Patients with residual or recurrent CIN2+ or VAIN2+ lesion were referred for a second treatment.

### 2.2. HPV Analyses

Search and typing of HPV DNA sequences was performed, as described in [18], by polymerase chain reaction (PCR) with consensus MY09/MY11 primers (which detect most high- and low-risk types); type identification was accomplished by restriction fragment length polymorphism (RFLP) analysis of MY amplimers, as well as PCR with HPV 16 type-specific 16H1/16R3 primers. DNA amplificability of all samples was verified by PCR with primers GH20/PC04 for the *β*-globin gene. For analysis of the HPV results, samples were classified as negative (no HPV DNA detected); positive for hrHPV types [19], with or without other types, differentiating HPV16 from the other hrHPV types; positive for low-risk (lrHPV) types (presence of any other HPV type).

### 2.3. Statistical Analyses

Cumulative incidence rates of residual and recurrent CIN2 and CIN3+ were computed by histological diagnosis at baseline (CIN2 versus CIN3+), overall (panel A: all 760 women treated for CIN2+ included in the study), and among women without residual high-grade disease at first follow-up visit, who had a hrHPV test (panel B: 506 women without residual high-grade disease at first follow-up visit and with HPV test). For statistical analyses, VAIN2 and VAIN3 cases were cumulated with CIN2 and CIN3+ cases, respectively.

The predictors of residual disease and/or recurrence risk (age at excision, pretreatment (initial) cytology, extension and number of lesions, vaginal involvement, and endocervical canal involvement at colposcopy) were assessed computing odds ratio (OR), with 95% confidence intervals (95% CI). Multivariate logistic regression models were used for each panel to predict the probability of a CIN2 or a CIN3+ event. All the parameters of interest were considered as covariates in the models, using a forward-stepwise selection to determinate which variables could be considered significant at *P* = 0.05.

## 3. Results

During the period between January 1, 2000, and December 31, 2009, a total of 810 women were treated for CIN2+ in our institution; 520 (64.2%) were attending the organized screening programme (target population: women 25–64 years old); 290 had been referred by other centres (women of any age). Among them, 20 moved in a distant area, 15 had less than 2 years of follow-up, and 15 did not attend any follow-up. Therefore, 760 women were included in our retrospective analysis; cases were classified according to the worst diagnosis, made either on the biopsy or the excised lesion. Of these, 415 had a diagnosis of CIN2, 330 of CIN3/in situ carcinoma, and 15 of microinvasive carcinoma (Table 1). Mean age at time of excision was 39 years (median 37.5; range 19–71); in particular, 287 women had <35 years (38%), 370 were in the 35–50 years age range (49%), and 103 were older than 50 years (13%).

LEEP was performed in 684 and conization in 76 cases, respectively; all but 15 (who requested general anesthesia for the presence of comorbidities) were treated in local anesthesia in an outpatient facility.

Histological evaluation of the excised specimens disclosed no lesion of any grade in 5 (6.6%) and CIN1 only in 116 (16%) cases. In all other cases, a high-grade lesion was confirmed. Margins status of the excised tissues were disease-free in 506 cases, positive in 126, not assessable in 20 (in 15 because of artefacts, and in 5 because no lesion was present in the excised specimen), while the data was unavailable in 108 (14%).

Median follow-up was 6.7 years (range 4–14; mean 7 years). Overall, during follow-up, 67 women (8.8%) were diagnosed with histologically confirmed CIN2+ or VAIN2+. The large majority of the lesions developed within the first two years of follow-up, at a higher rate after treatment of CIN3+ compared to CIN2 and with a plateau after two years only for cases treated for CIN3+.

### 3.1. Residual High-Grade Disease

The first posttreatment follow-up was performed after 3 months in women treated for microinvasive carcinoma and in most of the cases with positive margins and after 6 months in the remaining cases. Residual or progressive disease was detected at first follow-up visit in 39 women; 17 cases were diagnosed as CIN2, 15 cases were diagnosed as CIN3, 3 cases were diagnosed as microinvasive carcinomas, 1 case was diagnosed as invasive carcinoma (this was a FIGO stage 1A2 and occurred in an immunodepressed woman treated for CIN3, with positive endocervical margins, and subsequently treated by hysterectomy), 2 cases were diagnosed as VAIN2, and 1 case was diagnosed as VAIN3. In 6 cases, a diagnostic biopsy or a new excision was performed directly; in all other cases, the cytology was positive and guided the diagnostic work-up. The colposcopic impression was normal in 14/39 (36%) cases; 5 of these women had a type 3 TZ, and in 2 cases a VAIN2 was detected.

### 3.2. Recurrent High-Grade Disease

Among the 721 remaining cases, recurrent high-grade disease was found during subsequent follow-up in other 28 women (17 CIN2, 5 CIN3, 5 VAIN2, and 1 VAIN3; no invasive lesions occurred in this group). The colposcopic impression at the time of recurrence was normal in 9/28 (32%) cases; 5 of these women had a type 3 TZ, and in 3 cases a VAIN2 was detected. After 7 years of FU, the cumulative incidence rate for CIN3 was 6.7% among women treated for CIN3+ and 4.3% among women treated for CIN2; the cumulative incidence rate for CIN2 was 0.7% and 6.3%, respectively.

### 3.3. Predictors of Disease

Among the parameters analyzed as predictors of overall residual or recurrence risk, pretreatment (initial) cytology, extension and number of lesions, vaginal involvement, and endocervical canal involvement at colposcopy did not show any statistically significant correlation.

A higher CIN2+ risk in women with increasing age and with not fully visible junction at the time of treatment was observed, but the associations are statistically not significant.

Instead, the risk of posttreatment CIN2+ occurrence was higher in women with an original G2 colposcopic diagnosis (OR versus G1 2.55, 95% CI 1.28–5.08) and in cases with positive margins (OR 8.04, 95% CI 4.31–15.0). Moreover, the risk of residual or recurrent CIN3+ was higher in women whose original lesion was CIN3+ (OR 6.02, 95% CI 1.73–20.9) (Table 2).

**Figure 2:**
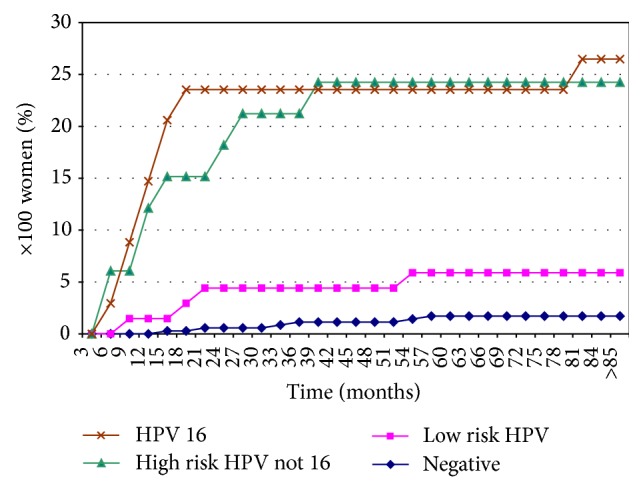
Cumulative incidence rates of recurrent CIN2+, by result of HPV typing at first FU after treatment and time since excision. Only women without residual high-grade disease at first follow-up visit, who had an HPV test result (panel B) are included.

HPV testing at 6–12 months after treatment was added from 2005 and gradually implemented; it was available for a minority of the 39 cases with residual disease (4/4 positive for hrHPV) and for 506 out of the 721 cases (70%) without residual high-grade lesions. No HPV DNA sequences were detected in 350 cases (69%), while HPV typing of the 156 HPV-positive cases disclosed a high-risk type in 67 (13%; HPV16 in 34, and other hrHPV types in 33 cases) and a low-risk type in 89 (18%) cases. Recurrent lesions (17 CIN2, 5 CIN3, 5 VAIN2, and 1 VAIN3) developed almost exclusively among women who had a positive hrHPV test at 6–12-month follow-up (Figure 2, panel B). The cumulative incidence rate of recurrent CIN2+ was steeper in HPV16+ cases, reaching a plateau 18 months after treatment, compared to 36 months in those with other hrHPV types. The cumulative CIN2+ incidence rates at the end of the follow-up were 26.5% in HPV16+ cases and 24.2% in other hrHPV+ cases; the corresponding figures for CIN3 were 8.8% and 3.0%, respectively. Among women positive for a low-risk HPV type at 6–12-month follow-up, a recurrent CIN2 lesion occurred in 4 cases, with a cumulative incidence rate of 5.9%.

Cytology was regularly performed throughout follow-up. It was repeatedly negative in more than half of the women, among whom 1 single CIN2 lesion occurred (0.25%). It was abnormal (ASC-US or worse) at least once in the others; this prompted additional interventions, and the frequency of detection of high-grade lesions was proportional to the degree of the cytological abnormality, ranging from <5% for ASC-US and LSIL (low-grade squamous intraepithelial lesion) to almost 80% for HGSIL (high-grade squamous intraepithelial lesion).

Among the 721 women without residual disease (panel B), the overall risk of recurrent CIN2 or CIN3 was correlated only with infection with HPV16 (OR 20.6, 95% CI 6.8–62.6) or other hrHPV types (OR 18.3, 95% CI 5.9–57.0). Table 2 shows the parameters associated with the recurrence of CIN2 and CIN3 separately. Besides HPV status at 6–12-month follow-up, recurrence of CIN2 was higher in women with a G2 colposcopic diagnosis (OR 4.17, 95% CI 1.28–13.6) and in those with an original histological diagnosis of CIN2 (statistically not significant). The risk of recurrent CIN3 was associated with HPV status at 6–12-month follow-up (OR 63.6, 95% CI 4.45–900, for HPV16 positivity) and type 3 squamocolumnar junction at baseline (OR 27.7, 95% CI 2.07–369).

## 4. Discussion

This is a retrospective observational study on 760 women treated for histologically confirmed CIN2+ lesions in a single institution and followed-up for a median of 6.7 years (range 4–14). Aim of the study was to assess their long-term risk of developing (new) high-grade lesions. Overall, the residual/recurrence rate was 8.8%; 5.1% were detected at first follow-up (residual disease) and 3.7% after a negative follow-up (recurrent disease). Progressive disease was recorded in 4 cases (0.5%), all of which were after CIN3(+) and within the first year after treatment: 1 invasive carcinoma (hysterectomy performed 10 months after conization) and 3 microinvasive carcinomas. No cases of invasive squamous or adenocarcinoma were recorded during the long-term follow-up. The most used treatment modality was loop electrosurgical excision procedure (LEEP), personalized and performed by highly experienced personnel; the 8.8% rate of residual/recurrent high-grade lesions was at the lower end of the 5–30% published rates [4, 20]. The risk to develop invasive cervical disease in women treated for a high-grade lesion has been reported to persist for many years [5, 6, 21]. In a recent cohort study covering the whole Swedish population for half a century [7], the risk of developing or dying from cervical or vaginal cancer in women treated for CIN3 was two to three times higher than that in the general female population, and the risk increased with increasing age at treatment and with ageing of treated women. In our study group, no invasive disease was detected after the first year after treatment, and only a (statistically not significant) trend for higher risk of CIN2/CIN3 recurrence in older women was observed. Our study is completely different for design, number of women evaluated, and length of follow-up and no direct comparison of the two studies can be made.

The identification of biomarkers predictive of precancer and cancer development after excisional treatment is important to modulate the follow-up in order to guarantee high sensitivity for detecting recurring lesions and to avoid excessive controls of cured women. Follow-up protocols show some differences among different countries and are subject to additional changes as a result of the technological innovations and the new cervical cancer preventive strategies (i.e., HPV-based screening, HPV vaccination, and new biomarkers) [22, 23].

Positive margins of the excised lesion were predictive for both residual and recurrent CIN2+ lesions, with higher OR for CIN3+ compared to that for CIN2, a result comparable to what is reported in another study of women treated for high-grade lesions [17] and in a study of women treated for stage Ia1 squamous cervical cancer [24].

Among the baseline anatomoclinical characteristics analyzed as long-term predictors of recurrent disease, statistically significant ORs were found for type 3 SCJ, G2 colposcopic diagnosis, and CIN3+ histological diagnosis; interestingly, they partly differed for CIN2 and CIN3 recurrence.

G2 as original colposcopic diagnosis was a significant predictor for recurrent CIN2 but not for CIN3; this parameter was dropped by our model as nonsignificant, probably because of the existence of stronger predictors for CIN3. CIN3+ as original diagnosis was predictive of CIN3+ recurrence.

Colposcopy represents a crucial step in the management of women with abnormal screening tests, since it is the method used to identify the type and features of the transformation zone and determines the reliability of the diagnostic biopsies, but conflicting results have been reported on its accuracy [25]. In our study, a type 3 TZ was associated with a quite high odds ratio for CIN3 posttreatment development. This is a rare occurrence, more often observed among older women; in our study, it was recorded in 7% of the 760 treated women, but it was present in 15% (10/67) of the cases with residual/recurrent lesions. Both experience and ability of the colposcopist influence the capacity to detect and sample a lesion, particularly when it is small in size; increasing the number of biopsies has been suggested as a potential way to improve colposcopy sensitivity [26], but this could eventually represent an overdiagnosis [27]. Indeed, HPV-based screening and HPV prophylactic vaccination will likely modify the frequency and extent of cervical abnormalities in the future; this might adversely affect colposcopic performance, and efforts to improve its quality assurance are of utmost importance [28].

A positive hrHPV test at first follow-up (6–12 months after treatment) was found in 13% of the cases with available data without residual disease and was positively associated with recurrent disease. Moreover, positivity for HPV 16 was associated with a very high OR for CIN3. Posttreatment hrHPV testing at 6-month follow-up has been clearly demonstrated to have higher sensitivity than cytology and comparable specificity [8, 11, 12], while a potential value of genotyping has been suggested [13]. Indeed, HPV 16 is known to have a higher oncogenic capacity than the other known high-risk types [29], with implications for natural history (faster development and higher persistence rate) and management [30].

The distribution over time of the residual/recurrent high-grade lesions showed that most of them developed within 2 years, as already reported in the literature [4], but disclosed some differences between CIN2 and CIN3. In particular, while a plateau was always observed for CIN3 lesions (irrespective of the grade of the initial lesion) and CIN2 after CIN3+, CIN2 following CIN2 showed a constant distribution over time. Indeed, a debate is ongoing on whether CIN2 is a definite entity and a truly intermediary step or the result of misclassification of CIN1 and CIN3 [3]; its diagnostic reproducibility is very low and its regression rate is rather high, especially in young women. Moreover, differences in risk factors, showing that CIN3 is more similar to cancer than CIN2, have been recently highlighted [31]. Our data show that CIN2 recurred mostly as CIN2, with a specific temporal behaviour, and biomarkers for CIN2 recurrence partly differed from those for CIN3. It is known that clinical outcome of an HPV infection is the result of a complex balance between immune system responses and viral immune evasion mechanisms; our data suggest that CIN2 (particularly when associated with a non-HPV16 type) may represent a persistent poorly controlled HPV infection rather than a true preneoplastic lesion.

The strengths of our study are the large number of patients followed up for much more than two years and treated in the same institution by experienced personnel, in a routine setting. Long-term evaluation is particularly important to understand the real risk of progressive disease in women treated for CIN2+, and homogeneity in treatment modality minimizes the differences present when analyzing multicentre cohorts.

The limitations of our study are mainly represented by the retrospective nature of data analysis, the late use of HPV testing and the missing results for some of the analyzed parameters; nonetheless, these weaknesses reflect what occurs in the every-day routine clinical setting.

Although our study is not powered to give information that may immediately translate into a modification of the follow-up protocols, the long-term clinical outcome and the results on the analyzed biomarkers allow some considerations and practical applications. Accurate definition of the type of transformation zone, the histological grade, and the margins status of the excised lesion appear to be very important; closer follow-up is necessary in case of type 3 TZ, CIN3+ lesion, and positive margins. Accuracy and experience of the personnel performing the excision also play a major role, stressing the need for training and quality assurance. Testing for hrHPV (with eventually partial typing for HPV16) 6–12 months after treatment is very effective in discriminating the women at higher and lower risk of recurrence.

The role and frequency of colposcopy after treatment are a matter of debate also in Italy [32]. While it has been performed in all women of our study group (treated between 2000 and 2009) at 6- and 12-month posttreatment follow-up visits, most recent guidelines (i.e., United Kingdom [33]) are not recommending colposcopy as routine practice in posttreatment follow-up. Indeed, while the colposcopy at 12 months after excision is deemed not necessary [34] (no longer performed in our institution), the colposcopy at first follow-up visit could be useful to localize the new SCJ, and as autoverification on the quality of the excision procedure.

## 5. Conclusions

Our retrospective analysis to assess the risk of high-grade CIN in women treated for CIN2+ lesions in a single institution on a routine basis and followed up for up to 14 years shows a favourable long-term clinical outcome in the great majority. Progressive disease was detected only in the first year after treatment. The type of transformation zone, the lesion grade, the status of the margins, and the result of hrHPV test at 6–12-month follow-up are the most useful parameters to predict long-term treatment outcomes.

## Figures and Tables

**Table 1:** Principal characteristics of the 760 cases included in the study.

	*N*	%
Histological diagnosis at baseline		
CIN2	415	54.6
CIN3/in situ carcinoma	330	43.4
Microinvasive carcinoma	15	2.0

Colposcopic diagnosis at baseline		
Negative	87	11.4
G1 (abnormal grade 1)	378	49.7
G2 (abnormal grade 2)	279	36.7
Suspected invasive carcinoma	1	0.1
Missing	15	2.0

Squamocolumnar junction location		
Type 1 (visible, ectocervical)	532	70.0
Type 2 (visible, endocervical)	158	20.8
Type 3 (nonvisible, in endocervix)	52	6.8
Missing	18	2.4

Treatment		
LEEP	684	90.0
Conization	76	10.0

Margins status of the excised specimens		
Negative	506	66.6
Positive	126	16.6
Not assessable	20	2.6
Missing	108	14.2

HPV test at first follow-up		
Negative	350	48.5
HPV 16	34	4.7
Other high-risk HPV	33	4.6
Low-risk HPV	89	12.2
Not performed	215	29.8

Residual^*^ lesions		
CIN2	17	2.2
CIN3	15	2.0
Microinvasive carcinoma	3	0.4
Invasive carcinoma	1	0.1
VAIN2	2	0.3
VAIN3	1	0.1
Recurrent^**^ lesions		
CIN2	17	2.2
CIN3	5	0.7
VAIN2	5	0.7
VAIN3	1	0.1

^*^Residual: lesions detected at first follow-up.

^**^Recurrent: lesions detected after one or more negative follow-up visits.

**Table 2:** Multivariate logistic regression analyses to identify predictive factors of the risk of residual or recurrent high-grade lesions in 760 women treated for CIN2+ (upper part) and among 506 women without residual high-grade disease at first follow-up visit who had a HPV test (lower part). Only variables that resulted significant after a forward-stepwise selection are reported.

*Women treated for CIN2+* (*n* = 760)				
Variable	Risk of residual or recurrent CIN2		Risk of residual or recurrent CIN3+	
	Odds ratio^∧^ (95% CI)	*P* value	Odds ratio^∧^ (95% CI)	*P* value
Histological diagnosis at baseline				
CIN2^*^	§		1.00	—
CIN3+			6.02 (1.73–20.9)	0.005
Margins status of the excised lesions				
Negative^*^	1.00	—	1.00	—
Positive	5.11 (2.42–10.8)	<0.001	13.8 (4.98–38.5)	<0.001

*Women without residual high-grade disease at first follow-up visit with HPV test* (*n* = 506)				
Variable	Risk of recurrent CIN2		Risk of recurrent CIN3	
	Odds ratio^∧^ (95% CI)	*P* value	Odds ratio^∧^ (95% CI)	*P* value

Histological diagnosis at baseline				
CIN2^*^	1.00	—	§
CIN3+	0.4 (0.12–1.29)	0.125	
Squamocolumnar junction location				
Type 1^*^	§		1.00	—
Type 2			1.68 (0.14–20.5)	0.68
Type 3			27.7 (2.07–369)	0.012
Colposcopic diagnosis at baseline				
G1^*^	1.00	—	§
G2	4.17 (1.28–13.6)	0.018	
Negative	1.67 (0.37–7.61)	0.503	
HPV test at first follow-up				
Negative^*^	1.00	—	1.00	—
HPV 16	13.3 (3.48–50.5)	<0.001	63.6 (4.45–909)	0.002
Other high-risk HPV	22.3 (5.69–87.3)	<0.001	7.76 (0.42–142)	0.168
Low-risk HPV	2.94 (0.67–12.8)	0.152	—^†^	—

^*^Reference.

^∧^Adjusted for all the variables in the table.

^§^Nonsignificant, excluded from the model.

^†^No CIN3+ events in the Low-risk group, excluded from the model.
